# Characterization of the complete chloroplast genome of *Leycesteria formosa* wall. (Caprifoliaceae), a medicinal plant in southwest China

**DOI:** 10.1080/23802359.2019.1704652

**Published:** 2020-01-10

**Authors:** Haizhu Zhang

**Affiliations:** a College of Pharmacy and Chemistry, Dali University, Dali, China;; b Key Laboratory of Yunnan Provincial Higher Education Institutions for Development of Yunnan Daodi Medicinal Materials Resources, Yunnan, China

**Keywords:** *Leycesteria formosa*, chloroplast, Illumina sequencing, phylogeny

## Abstract

*Leycesteria formosa* is a frequently used medicinal plant (namely, ‘Yi yao’) in Southwest China. In this study, we sequenced the complete chloroplast (cp) genome sequence of *L. formosa* to investigate its phylogenetic relationship in the family Caprifoliaceae. The chloroplast genome of *L. formosa* was 156,261 bp in length with 38.1% overall GC content, including a large single-copy (LSC) region of 90,353 bp, a small single-copy (SSC) region of 19,299 bp and a pair of inverted repeats (IRs) of 23,287 bp. The cp genome contained 112 genes, including 79 protein-coding genes, 29 tRNA genes, and 4 rRNA genes. The phylogenetic analysis indicated *Leycesteria* was closely related to the genus *Triosteum* or *Heptacodium*.

*Leycesteria* is a genus of the Caprifoliaceae family, which are deciduous shrub or small shrub plants, includes 8 species all over the world. Most of the species are widespread in the Himalayas and Myanmar. There are six species in China, mainly distributed in the temperate and subtropical mountains of southwest China (The Editorial Committee of Flora of China 1988). Some species in this genus have been widely used in traditional Chinese medicine for thousands of years. Among these species, *L. formosa* is an original species of famous traditional Chinese medicine. *Leycesteria formosa* is also known as the gun barrel, hollow wood, wild lupine, golden chicken, lock, etc. In southwest China, *L. formosa* has the function of dampness and heat removal, promoting blood circulation and stop bleeding. It is used as the alternative of genuine medicine for the treatment of damp heat jaundice, rheumatic pain, asthma, irregular menstruation, traumatic bleeding, cystitis, fracture damage, etc. (The Editorial Committee of Pharmaceutical Administration of China 1999). However, until now, most of the studies for this species mainly focused on describing its chemical compositions (Lobstein et al. 2002; He 2014), with little involvement in its molecular biology. Here, we reported the complete chloroplast genome sequence of *L. Formosa* and revealed its phylogenetic relationships with other species in the Caprifoliaceae.

Fresh leaf materials of *L. formosa* were sampled from Dali County, Yunnan, China (N25°52′33.75″, E100°1′51.23″); meanwhile, a voucher specimen (No. ZDQ17069) was collected and deposited at the Herbarium of Medicinal Plants and Crude Drugs of the College of Pharmacy and Chemistry, Dali University. The total genomic DNA was extracted using the improved CTAB method (Doyle 1987; Yang et al. 2014) and sequenced with Illumina Hiseq 2500 (Novogene, Tianjing, China) platform with pair-end (2 × 300 bp) library. About 3.17 Gb of raw reads with 10,524,674 paired-end reads were obtained from high-throughput sequencing. The raw data were filtered using Trimmomatic v.0.32 with default settings (Bolger et al. 2014) and were assembled into circular contigs using GetOrganelle.py (Jin et al. 2018). Finally, the cpDNA was annotated by the Dual Organellar Genome Annotator (DOGMA; http://dogma.ccbb.utexas.edu/) (Wyman et al. 2004) and tRNAscan-SE (Lowe and Chan 2016).

The annotated chloroplast genome was submitted to the GenBank under the accession number MN755836. The total length of the chloroplast genome was 156,261 bp, with 38.1% overall GC content. With typical quadripartite structure, a pair of IRs (inverted repeats) of 23,287 bp was separated by a small single copy (SSC) region of 19,299 bp and a large single copy (LSC) region of 90,353 bp. The cp genome contained 112 genes, including 79 protein-coding genes, 29 tRNA genes, and 4 rRNA genes. Among these genes, 15 genes were duplicated in the inverted repeat regions, 10 genes, and 6 tRNA genes contain one intron, while two genes (*ycf3* and *rps18*) have two introns.

To investigate its taxonomic status, a total of 37 cp genome sequences of Caprifoliaceae species were downloaded from the NCBI database used for phylogenetic analysis. After using MAFFT V.7.149 for aligning (Katoh and Standley 2013), a neighbor-joining (NJ) tree was constructed in MEGA v.7.0.26 (Kumar et al. 2016) with 1000 bootstrap replicates and two Caprifoliaceae species (*Sambucus williamsii*: MN524614 and *Sambucus nigra*: MN524613) were used as outgroups. The results showed that *Leycesteria* was closely related to the genus *Triosteum* or *Heptacodium* (Figure 1). Meanwhile, the phylogenetic relationship in Caprifoliaceae was consistent with previous studies and this will be useful data for developing markers for further studies.

**Figure 1.**
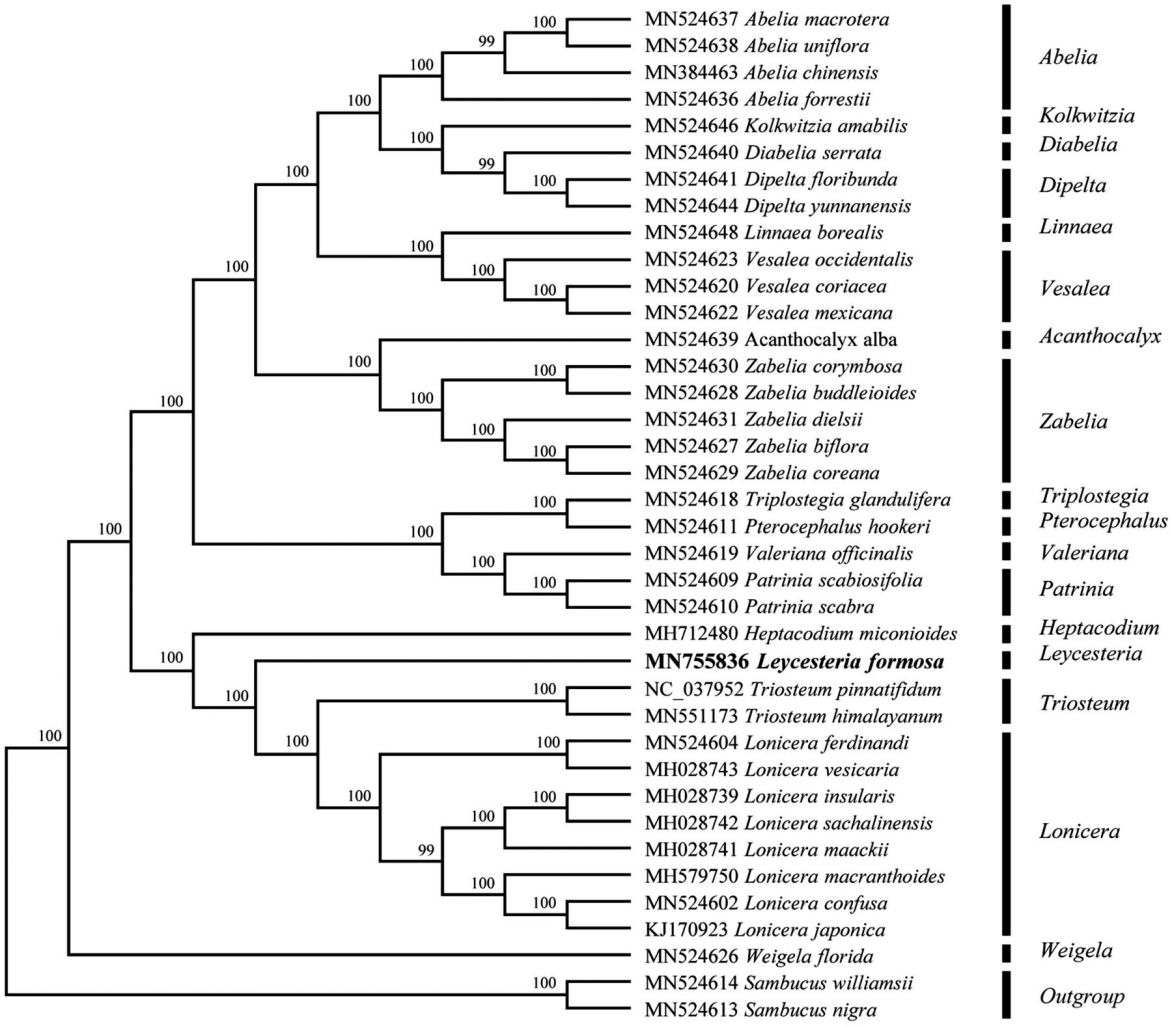
Neighbor-joining (NJ) tree of 38 species within the family Caprifoliaceae based on the plastomes using two Caprifoliaceae species as outgroups.
